# Ab-GALFA, A bioassay for insect gall formation using the model plant *Arabidopsis thaliana*

**DOI:** 10.1038/s41598-023-29302-8

**Published:** 2023-02-13

**Authors:** Tomoko Hirano, Ayaka Okamoto, Yoshihisa Oda, Tomoaki Sakamoto, Seiji Takeda, Takakazu Matsuura, Yoko Ikeda, Takumi Higaki, Seisuke Kimura, Masa H. Sato

**Affiliations:** 1 Graduate School of Life and Environmental Sciences, Kyoto Prefectural University, Shimogamo-Hangi-Cho, Sakyo-Ku, Kyoto, 606-8522 Japan; 2 Center for Frontier Natural History, Kyoto Prefectural University, Shimogamo-Hangi-Cho, Sakyo-Ku, Kyoto, 606-8522 Japan; 3 Department of Biological Science, Graduate School of Science, Nagoya University, Furo-Cho, Chikusa-Ku, Nagoya, Aichi 464-8602 Japan; 4 Laboratory of Plant Ecological and Evolutionary Developmental Biology, Department of Bioresource and Environmental Sciences, Kyoto Sangyo University, Kamigamo-Motoyama, Kita-Ku, Kyoto, 603-8555 Japan; 5 Biotechnology Research Department, Kyoto Prefectural Agriculture, Forestry and Fisheries Technology Center, 74 Oji, Kitainayazuma, Seika-Cho, Soraku-Gun, Kyoto, 619-0244 Japan; 6 Institute of Plant Science and Resources, Okayama University, Chuo 2-20-1, Kurashiki, Okayama 710-0046 Japan; 7 Department of Biological Sciences, Graduate School of Science and Technology, Kumamoto University, Kumamoto, 860-8555 Japan

**Keywords:** Biological techniques, Molecular biology, Plant sciences

## Abstract

Insect galls are abnormal plant organs formed by gall-inducing insects to provide shelter and nutrients for themselves. Although insect galls are spatialized complex structures with unique shapes and functions, the molecular mechanism of the gall formation and the screening system for the gall inducing effectors remains unknown. Here, we demonstrate that an extract of a gall-inducing aphid, *Schlechtendalia chinensis*, induces an abnormal structure in the root-tip region of *Arabidopsis* seedlings. The abnormal structure is composed of stem-like cells, vascular, and protective tissues, as observed in typical insect galls. Furthermore, we confirm similarities in the gene expression profiles between the aphid-treated seedlings and the early developmental stages of *Rhus javanica* galls formed by *S. chinensis*. Based on the results, we propose a model system for analyzing the molecular mechanisms of gall formation: the *Arabidopsis*-based Gall-Forming Assay (Ab-GALFA). Ab-GALFA could be used not only as a model to elucidate the mechanisms underlying gall formation, but also as a bioassay system to isolate insect effector molecules of gall-induction.

## Introduction

Galls on plants are abnormal organs formed by various plant parasites including viruses, fungi, bacteria, nematodes, and insects. Insect galls have extremely complex and highly organized structures comprising several specialized tissues that the insects utilize for shelter and nutrients^1,2^. Many remarkable flower- and fruit-like structures have been observed in insect galls, particularly those induced by gall midges, aphids, and cynipids in various host plant species^3^, suggesting that the formation of gall tissues is similar to that of flowers or fruits^4,5^.

We have previously performed RNA-sequencing-based comparative transcriptomic analysis of the early developmental stage of horned gall to elucidate the early gall-inducing process carried out by a gall-inducing aphid, *Schlechtendalia chinensis*, in the Chinese sumac, *Rhus javanica*, and found that several reproductive organ genes are expressed in the early development phase of galls induced^6^. Furthermore, the comparative study of three-different galls induce on *Artemisia montana*, *Glochidion obovatum*, and *R. javanica* indicated that several key regulators of stem cell generation (CLE44, BAM3, WOXs) and maintenance floral organ development (SEPALLATA, AGAMOUS, and APETALA1) were commonly upregulated during gall development^7,8^*.*

In the plant tissues level, insect galls include (i) callus-like cells transformed from palisade and spongy tissues used for insect feeding in inner-layer cells (ii) protective outer tissue composed of lignified secondary cell walls for shelter and protection in outer-layer cells, and (iii) vascular tissues which transports water and nutrients to the callus-like cells in the gall^9–11^.

To generate these complex gall structures, gall-inducing insects are believed to manipulate plant developmental programs by secreting effector molecules^2,12,13^. In fact, phytohormones, including significant quantities of auxins and cytokinins, as well as certain amino acids and proteins, have been detected in gall-inducing insects^2,14–18^. Such chemical stimuli are produced via the salivary secretions and oviposition fluids produced by larvae, parent insects, or both^13–15^.

Thus, gall-inducing insects secrete effector molecules into plant tissues using their mouthparts or ovipositors to manipulate plant development, thereby generating complex gall structures in host plants^1,2,13^. However, owing to the lack of model symbiotic systems for dissecting the molecular mechanisms of insect gall formation, the effector molecules and mechanisms underlying the gall formation process in plants remain largely unknown.

We hypothesize that the effectors control the developmental pathways in the model plant *A. thaliana*, as well as in the host plants, since the molecular machinery for development and the developmental pathways are highly conserved among higher plants.

Here, we develop the *Arabidopsis*-based Gall Formation Assay (Ab-GALFA), an effective method of analyzing the molecular mechanisms underlying gall formation using a model plant, *Arabidopsis thaliana*. This model system could be used to elucidate the molecular basis for gall formation in plants, and can be utilized as a bioassay for isolating the unknown insect effectors of gall formation in various host plants.

## Results

### Extract of a gall-inducing insect can alter the morphology of the model plant, *A. thaliana*

**Figure 1:**
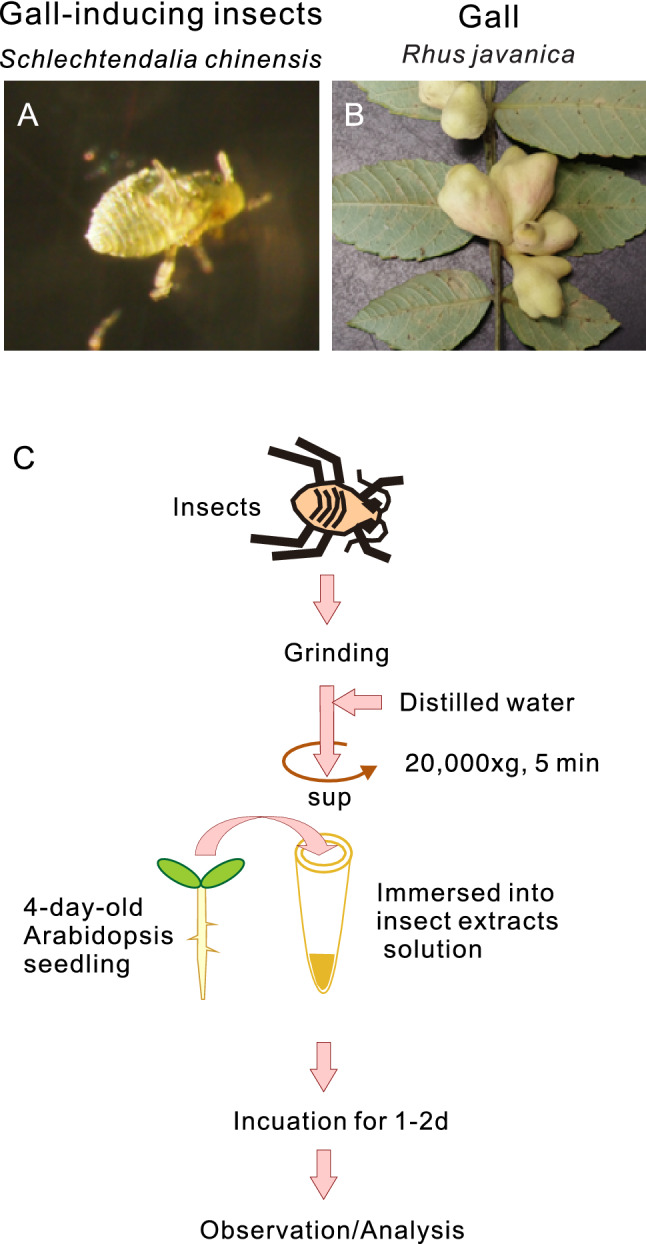
Schematic illustration explaining the use of Ab-GALFA for testing the gall-inducing effect of an aphid, *Schlechtendalia chinensis*. *S. chinensis* (**A**) induces large, single-chamber galls (**B**) in *Rhus javanica.* (**C**) Insect bodies are ground in liquid nitrogen and suspended in sterile distilled water to prepare the insect extract solution. *Arabidopsis* seedlings incubated in the solution are observed or analyzed via confocal microscopy or RNA sequencing.

We determined whether treating the seedlings of the model plant, *A. thaliana*, with an extract of a gall-inducing insect would induce morphological changes in the *Arabidopsis* tissues. As a first attempt, we tested *S. chinensis* as a model gall-inducing insect for the following reasons: (i) *S. chinensis* induces large gall over a short period, (ii) a large volume of clone insect bodies could be available in one gall tissue, (iii) we have previously investigated the expression profile of the early stage of gall (Fig. 1A,B)^6^.

**Figure 2:**
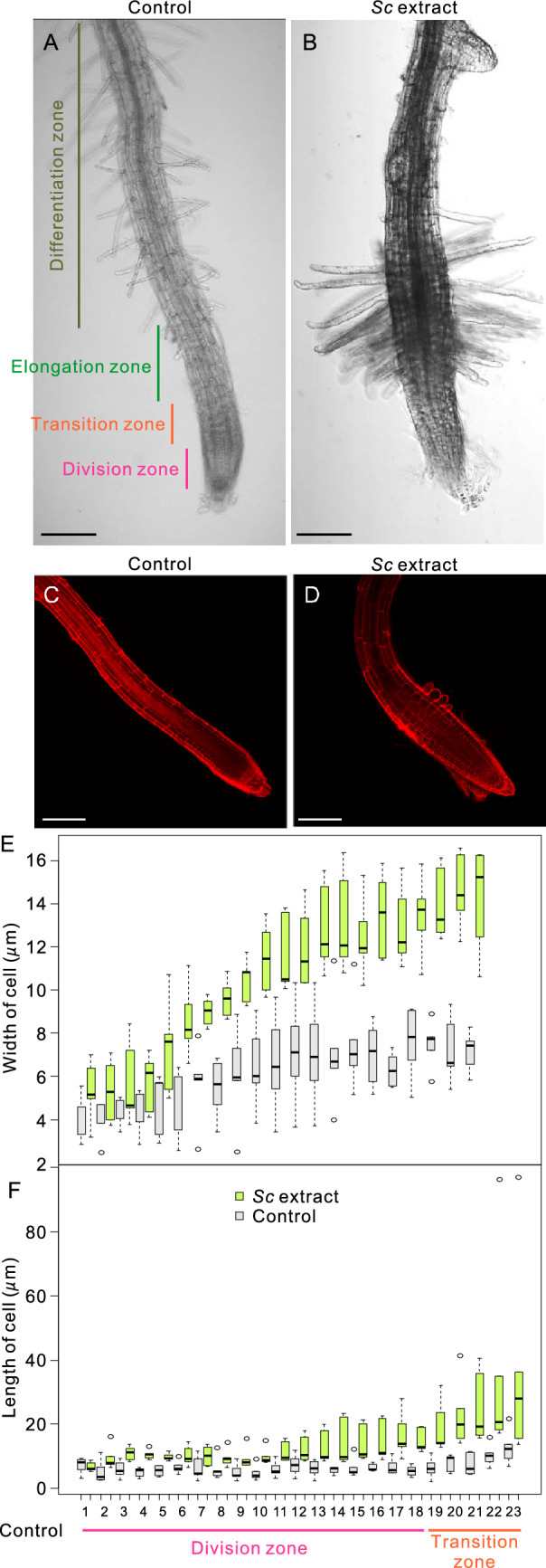
Morphological changes in the root-tip region of *Arabidopsis* after *S. chinensis* extract treatment. The morphology of the root-tip region of *Arabidopsis* seedlings without (**A**) or with (**B**) *S. chinensis* extract treatment*.* Propidium iodide-stained root tips of *Arabidopsis* seedlings without (**C**) or with (**D**) *S. chinensis* extract treatment*.* The length (**E**) and width (**F**) of the root epidermal cells were measured from the initial root epidermal cell. Numbers represent the cell number from the initial cell. Blue: control, orange: after *S. chinensis* extract treatment*.* Asterisks denote significant differences based on the Mann–Whitney *U*-test (*p* < 0.05); n = 3 biological replicates of five seedlings each. Scale bars: 100 µm (**A**–**D**).

Firstly, morphological changes in 4-day-old *Arabidopsis* seedlings soaked in various concentrations of the extract of gall-inducing aphid, *S. chinensis*, (hereafter referred to as *Sc* extract) were observed via light microscopy after 1 day of treatment (Fig. 1C)*.* We found that several morphologies of the seedlings were significantly changed. For example, the root and elongation zones abnormally thickened, with several thick root hair-like structures that emerged from the root elongation zone (Fig. 2A and B, Supplemental Movie 1). These morphological changes were not observed following treatment with extracts of the non-galling aphid species *Acyrthosiphon pisum* (*A. p*) and *Megoura crassicauda* (*M. c*) (Fig. S1–S5), suggesting that the phenomenon was caused specifically by the extract of the gall-inducing insect.

We then examined the morphological changes that occur at the cellular level. We stained *Sc* extract-treated *Arabidopsis* seedlings with propidium iodide (Fig. 2C and D) and measured the vertical and horizontal lengths of the epidermal cells of the root-tip region. The width of the root epidermal cells from the division zone to the transition zone was increased, whereas the length of the root epidermal cells was only increased in the transition zone, when the seedlings were incubated with *Sc* extract. (Fig. 2E and F). From these results, we concluded that the morphological changes in the root-tip region induced by the the *Sc extract* were attributed to changes in the wider shape of the root epidermal cells from the division to the transition zones in the root.

**Figure 3:**
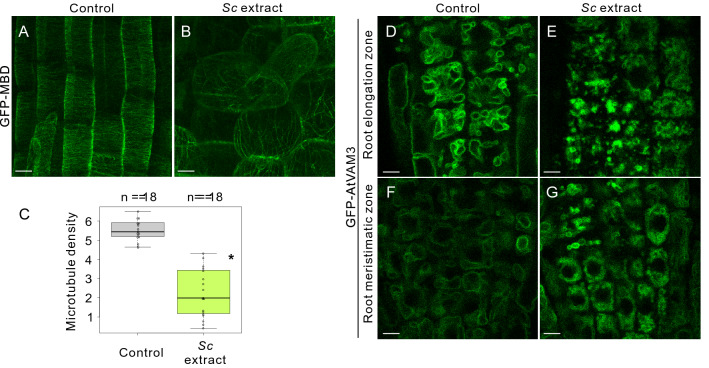
Changes in the structure of cortical microtubules and vacuoles in root epidermal cells after aphid extract treatment. (**A**–**C**) Live cell imaging of *Arabidopsis* root-transition zone expressing microtubule fluorescent markers GFP-MBD without (**A**) or with (**B**) *S. chinensis* extract treatment; (C) The density of cortical microtubules. The asterisk denotes a statistically significant difference between the control and *Sc* extract-treated conditions (*p < 0.05; Mann–Whitney *U*-test). Biological replicates of 18 seedlings. In the box-and-whisker plots, the boxes and solid lines in the boxes show the upper (75th) and lower (25th) quartiles and median values, respectively. The whiskers indicate the 95% confidence intervals. The experiments were independently repeated three times. (**D**–**G**) Live cell imaging of *Arabidopsis* root-transition zone and root division zone of transgenic *Arabidopsis* line expressing a vacuole membrane fluorescent marker GFP-AtVAM3 without (**D**, **F**) or with (E, G) *S. chinensis* extract treatment. Scale bars: 10 μm.

The morphological changes in the root epidermal cells following treatment with the *Sc* extract might have been caused by changes in the cytoskeleton and/or organelle structures. A cytoskeletal structure, cortical microtubules, and the most prominent plant organelle, the central vacuole, are important for the construction of cell shape in plants^19,20^. Therefore, we investigated the mechanism by which the morphology of the cortical microtubules and central vacuoles were altered by the *Sc* extract treatment. The parallel alignment of the cortical microtubule array visualized using a fluorescent microtubule marker, GFP-MBD^21^. Measurement of the microtubule density of root epidermal cells of root elongation zone indicated that the cortical microtubule array was severely disordered by the *Sc* extract treatment (Fig. 3A–C).

Plant vacuoles have a dynamic membrane system that undergoes complex architectural remodeling during developmental process^22,23^. In particular, dynamic morphological remodeling, in which fragmented small vacuoles are fused and form large central vacuolar structures, occurs during the development of mature root epidermal cells from root meristematic cells^24,25^. After treatment with the *Sc* extract, the bursiform and complex structures of the vacuoles in the root meristematic and elongation zones changed to more fragmented, smaller vacuolar structures (Fig. 3D–G) that resembled the vacuolar structures in root meristematic cells^24^.

### *S. chinensis* extract contains significant amounts of phytohormones

Gall-inducing insects are known to produce phytohormones in their bodies and inject them into host plants to induce gall-structure development^13,14,17,26–28^. The application of appropriate combinations and concentrations of exogenous phytohormones can induce gall-like structures in plants^28^. These observations imply that the gall-like structure that emerged in the root region following treatment with the the *Sc* extract might be caused by exogenous phytohormones from the aphid bodies. Therefore, we measured the concentrations of typical phytohormones in *S. chinensis* and considerable quantities of abscisic acid (ABA), salicylic acid (SA), jasmonic acid (JA), jasmonoyl-L-isoleucine (JA-Ile) were detected in addition to the presence of indole acetic acid (IAA), isopentyladenine (iP), and trans-zeatin (tZ) (Table 1), suggesting that various types of phytohormones can function as insect effector molecules for gall-induction.

**Table 1:** The exogenous phytohormones contents in *S.Chinensis*.

Phytohormones	Average (ng/g_FW)
IAA	676 ± 69.9
ABA	435 ± 75.8
SA	69.4 ± 24.0
JA	10.0 ± 3.91
iP	1.26 ± 0.11
JA-Ile	0.991 ± 1.41
tZ	0.915 ± 0.271

IAA, indole-3-acetic acid; ABA, abscisic acid; iP, isopentenyladenine; SA, salicylic acid; JA, jasmonic acid; JAIle, jasmonic acid-isoleucine; iP, isopentenyladenosine; tZ, trans-zeatin. Experiments were repeated at least three times using three biologically distinct samples.

The results are given as mean ± standard deviation from four biological replicates (approximately 50–100 mg per sample).

### Typical characteristics of insect gall-like structures that emerged in the root-tip region after the aphid extract and phytohormone mixture treatments

Typical insect galls consist of three representative tissues: stem cells, vascular, and protective tissues^1,2^. We tested whether these three representative gall tissues (stem cells vascular, and protective tissues) could be observed in the gall-like structures in the root region of the *Arabidopsis* seedlings after the treatment with the *Sc* extract or an artificial phytohormone mixture (AHM), which mimicked the content and concentration of the phytohormones in the *Sc* extract.

**Figure 4:**
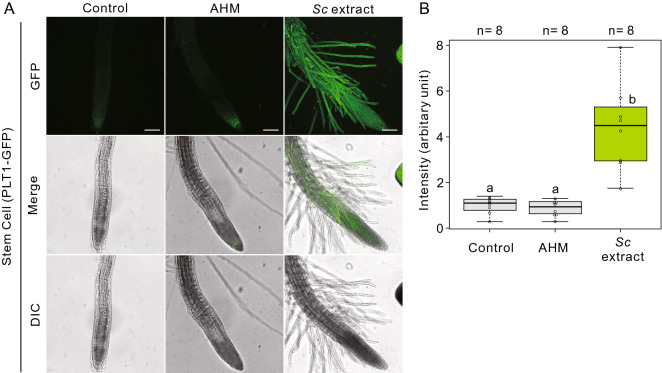
*S. chinensis* extract initialized epidermal cells of root elongation zone. (**A**) Fluorescence imaging of PLT1-GFP expressing lines without or with treatment using an artificial hormone mix (AHM) solution or *S. chinensis* extract. Scale bars = 100 µm. (**B**) Box-and-whisker plots showing the fluorescence intensity in (**A**) (n = 8 seedlings for each treatment, with three biological replicates). The boxes and solid lines in the boxes show the upper (75th) and lower (25th) quartiles and median values, respectively. Groups with different letters are significantly different from each other (*p* < 0.05, Wilcoxon and Steel–Dwass tests).

As previously reported, several key regulatory molecules in stem cell generation are expressed during the initial stages of gall formation^5–7^. Therefore, we used a stem cell niche marker, *PLETHORA1 (PLT1)*^29^, and a quiescent center (QC) marker, *WUSCHEL RELETATED HOMEOBOX (WOX5)*^30^ to determine whether the structure of the stem cell niche was altered following treatment with the *Sc* extract or AHM. PLT1-GFP fluorescence was also observed not only in the epidermal cells of the division zone but also of the root transition, elongation and division zones in the *Sc* extract treatment (Fig. 4). The ectopic expression of *PLT1* in the cells of the root-transition and elongation zone was not observed following treatment with the extracts of *A. pisum* and *M. crassicauda,* AHM, and a control treatment (water) (Fig. 4 and Fig. S1). A QC marker, *WOX5p::WOX5-GUS-mNeonGreen* exhibited similar expression pattern of the *PLT1p::PLT1-GFP* in root-tip region (Fig. S2).

These results indicated that the stem cell niche spread from the root-meristematic region to the root- elongation zones only after the *Sc* extract treatment.

**Figure 5:**
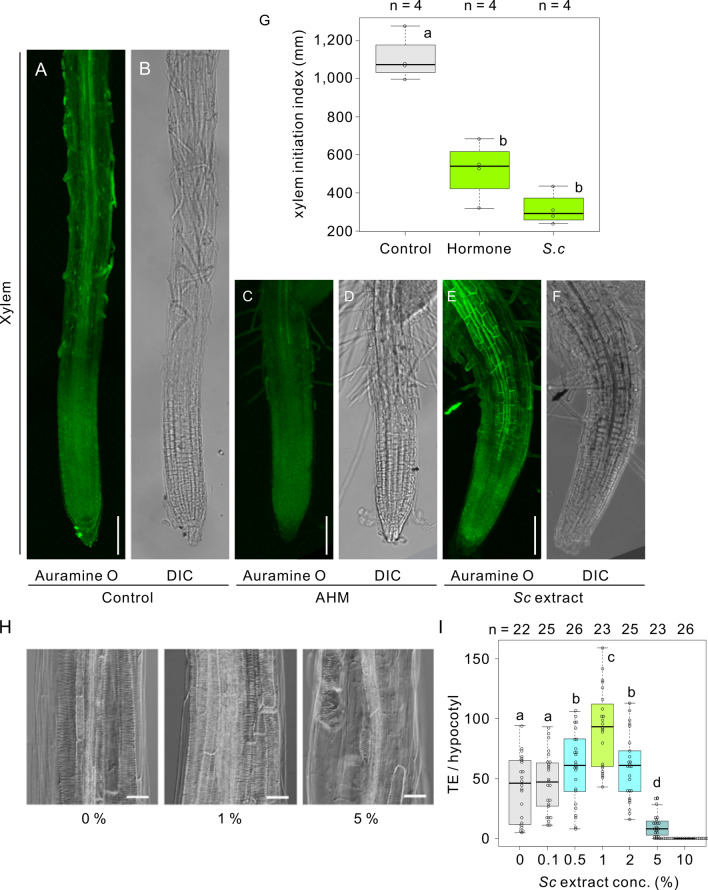
*S. chinensis* extract induced extended xylem tissue. Florescence image (**A**) and differential interference contrast (DIC) image (**B**) of Auramine O-stained root of control (DW) *Arabidopsis* seedling; florescence image (**C**) and DIC image (**D**) of Auramine O-stained root of artificial phytohormone mixture (AHM)-treated *Arabidopsis* seedling; florescence image (**E**) and DIC image (**F**) of Auramine O-stained root of *S. chinensis* extract (*Sc* extract)-treated *Arabidopsis* seedling. Scale bars = 100 µm. (**G**) Box-and-whisker plots showing the fluorescence intensity in (**A**) (n = 4 seedlings for each genotype, with three biological replicates). The boxes and solid lines in the boxes show the upper (75th) and lower (25th) quartiles and median values, respectively. Different letters represent significant differences in each point (*p* < 0.05, Wilcoxon and Steel–Dwass tests). (**H**) Effect of *S. chinensis* extract on TE differentiation in cultured hypocotyls cultured with *S. chinensis* extract. Scale bars = 50 µm. (**I**) TE differentiation rate. Data are presented as mean ± s.d. n > 20 hypocotyls; **p < 0.01 (ANOVA with Scheffe test).

Next, to investigate the effect of the *Sc* extract on xylem development, we stained the xylem structure of the *Sc* extract-treated *Arabidopsis* seedlings with Auramine O, a fluorescent dye that mainly stains lignin and suberin^31^. Under normal growth conditions, the distance from the root tip to the protoxylem tracheary elements was approximately 1000 µm, whereas the distance after AHM or the *Sc* extract treatment was shortened to ~ 590 µm and ~ 300 µm, respectively (Fig. 5A–G), indicating that both the AHM and the *Sc* extract have xylem development activity. Intriguingly, additional lignin deposition was observed in the endodermal cells in the root-transition zone after the *Sc* extract treatment (Fig. 5E,F).

To further confirm the effects of the *Sc* extract on xylem vessel formation, segments of the hypocotyl were cultured in xylem vessel-inducing medium^32^ containing a 0–10% concentrations of the *Sc* extract stock solution (tenfold dilution of the stock solution). The frequency of xylem vessel formation was elevated at a 1% *Sc* extract concentration, and was relatively inhibited at concentrations above 2%. The application of up to 5% *Sc* extract inhibited tracheary element induction, suggesting that the *Sc* extract can promote or inhibit xylem vessel formation in a dose-dependent manner (Fig. 5H and I).

**Figure 6:**
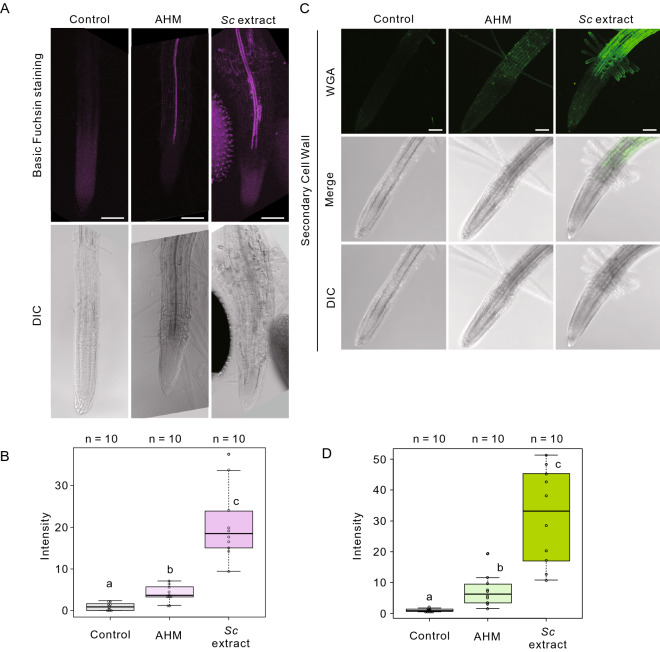
*S. chinensis* extract induced ectopic deposition of the secondary cell wall components in the epidermal cells of the root division zone. (**A**) Fluorescent and DIC images of 4-day-old *Arabidopsis* seedlings stained with Alexa Fluor 488-conjugated wheat germ agglutinin (WGA) for secondary cell wall labeling, without or with of the artificial hormone mix (AHM) or *S. chinensis* extract treatment. Scale bars = 100 µm. (**B**) Box-and-whisker plots showing the fluorescence intensity in (A) (n = 10 seedlings for each genotype, with three biological replicates). The boxes and solid lines in the boxes show the upper (75th) and lower (25th) quartiles and median values, respectively. Different letters in (**B**) represent significant differences in each point (*P* < 0.05, Wilcoxon and Steel–Dwass test). (**C**) Basic fuchsin staining of the roots of 4-day-old *Arabidopsis* seedlings without or with AHM solution or *S. chinensis* extract treatment. Scale bars = 100 µm. (**D**) Box-and-whisker plot showing the fluorescence intensity in (**C**) (n = 10 seedlings for each genotype, with three biological replicates). The boxes and solid lines in the boxes show the upper (75th) and lower (25th) quartiles and median values, respectively. Different letters in (**D**) represent significant differences in each point (*p* < 0.05, Wilcoxon and Steel–Dwass tests).

We also confirmed that basic fuchsin-stained lignified tissues, a component of the secondary cell wall, increased more than fourfold after the *Sc* extract treatment compared with after the AHM treatment (Fig. 6A and B). Notably, both protoxylem and metaxylem developed into the root elongation zone following the *Sc* extract treatment, whereas only protoxylem developed after AHM treatment, suggesting that the *Sc* extract has an ability to induce tracheary elements (Fig. 6B).

Finally, we determined whether the *Sc* extract could induce secondary cell wall in root-tip region. The secondary cell wall was stained with Alexa Fluor 488-conjugated wheat germ agglutinin (WGA), which binds to the 4-O-methylglucuronic acid of xylan and is an indicator of the secondary cell wall^32^. After the *Sc* extract treatment, secondary cell wall emerged in the epidermal cells of the root transition and elongation zone; they were not observed in the control, AHM, *A. pisum* or *M. crassicauda* extract treatments (Fig. 6C and D, Fig. S3). Furthermore, we investigated whether the distribution of phytohormones, auxin, and cytokines is altered after the aphid extract treatments using fluorescent reporter lines of auxin (*DR5rev::GFP*)^33^ and cytokines (*TCS::GFP*)^34^, respectively. Auxin, but not cytokines, was spread following the *Sc* extract treatment. No changes in the distribution of auxin and cytokines were observed after *A. pisum* or *M. crassicauda* extract treatments (Figs. S4 and S5).

Collectively, only the *Sc* extract treatment induced gall-like structures in *Arabidopsis* seedlings, comprising secondary cell wall formation in the epidermal cells, the enhancement of xylem cell formation, and the dedifferentiation of epidermal and cortical cells in the root region. In contrast, AHM treatment only enhanced protoxylem elongation, suggesting that effectors other than plant hormones act to induce stem cell niche maintenance, secondary cell wall formation, and xylem cell formation.

### RNA-seq analyses of aphid extract and hormone mixture treatments

**Figure 7:**
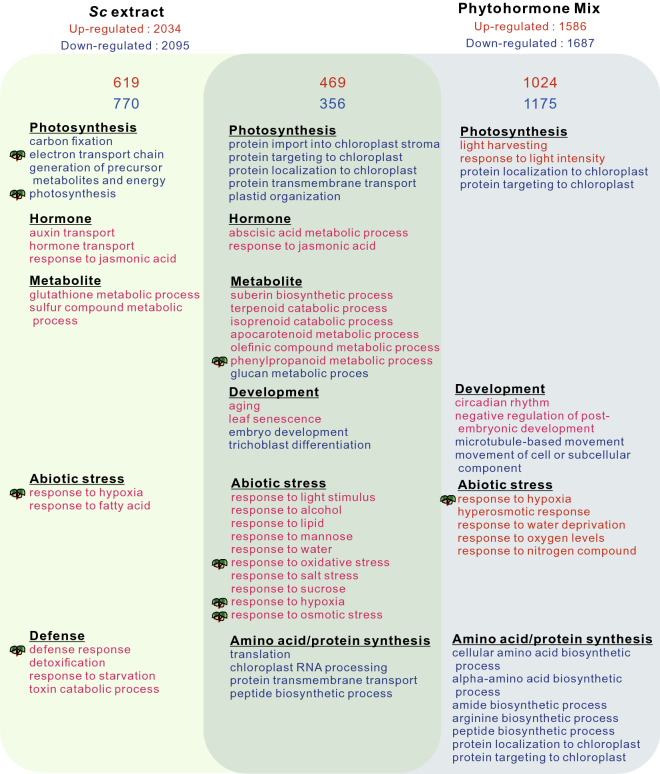
Venn diagram analysis of the number of increased differentially expressed genes (DEGs) in the *Arabidopsis* seedlings following *Schlechtendalia chinensis* extract treatment and phytohormone mixture treatment. The number in each circle represents the number of upregulated DEGs in each treatment. The number in the overlapping region indicates the shared DEGs between the two treatments. Descriptions in each region indicate the typically enriched gene ontology categories of the corresponding groups.  denotes the corresponding GO-term categories with the RNAseq analysis of the early developmental galls of *S. chinensis*^18^.

We performed RNA-seq analysis of *Arabidopsis* seedlings treated with the *Sc* extract and AHM to determine whether the changes in the expression of *Arabidopsis* seedlings are induced by only phytohormones or phytohormones plus other effector molecules in the *Sc* extract. The *Sc* extract and AHM treatments upregulated 4129 and 3273, differentially expressed genes (DEGs), respectively. A Venn diagram analysis indicated that of these DEGs, 619, 1,024, and 469 were upregulated in the *Sc* extract treatment, AHM treatment, and commonly upregulated in these treatments, respectively. In contrast, 770, 1,175, and 356 DEGs were downregulated in the *Sc* extract treatment, AHM treatment, and commonly downregulated in both treatments, respectively. We performed a gene ontology (GO) enrichment analysis on the commonly upregulated DEG sets in *Sc* and AHM treatments and found that the upregulated DEGs were categorized into Hormone (GO-term categories: “response to abscisic acid” and “response to jasmonic acid”), Abiotic stress response (GO-term categories: “response to light stimulus”, “response to alcohol”, “response to lipid”, “response to mannose”, “response to water”, “response to oxidative stress”, “response to salt stress”, “response to sucrose”, “response to hypoxia” and “response to osmotic stress”), Development (GO-term categories: “leaf senescence”, “aging”), Metabolite (GO-term categories: “suberin biosynthetic process”, “terpenoid catabolic process” “isoprenoid catabolic process”, “apocarotenoid metabolic process”, “phenylpropanoid biosynthetic process” and “olefinic compound metabolic process”) (Fig. 7, Supplementary Tables S1–S3). Among the 469 commonly upregulated genes, 57 transcription factors were differentially expressed. These transcription factors were categorized as auxin signal transduction, ABA signal transduction, and circadian rhythm (Table S4). As the *Sc* extract contained IAA and ABA, the upregulation of these transcription factors could be accomplished through the effects of aphid-derived phytohormones. The Ab-GALFA results revealed that the *Sc* extract treatment induced the dedifferentiation of the epidermal and cortical cells in the transition zone, secondary cell wall formation in the epidermal cells in the differentiation zone, and the enhancement of xylem cell formation in the root. In accordance with these morphological changes, transcriptional regulators of stem cell formation (CLE44, WOX5, and Class-1 KNOXs), xylem development (VND7), and secondary cell wall construction (MYB105, MYB42, MYB45, MYB52, MYB56, and MYB63) were significantly upregulated in the *Arabidopsis* seedlings treated with the *Sc* extract (Table 2).

**Table 2:**
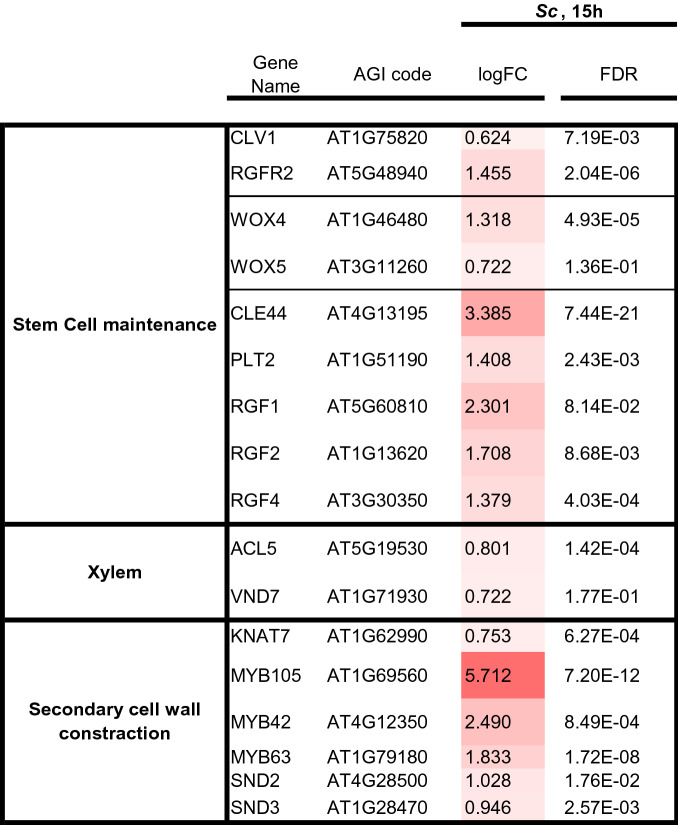
The genes associated with stem cell formation, xylem development, and the secondary cell wall were up-relulated in Arabidopsis seedlings treated with Sc extract or Hormone mixture, compared with in Rhus javanica.

Thus, although many genes were commonly upregulated in both the *Sc* extract and AHM treatments, the expression of significant number of genes were altered only in the *Sc* extract treatment (Fig. 7).

In the case of GO enrichment analysis of downregulated genes of *Arabidopsis* seedlings treated with *Sc* extract or AHM. The genes related to the photosynthetic process were downregulated both in the *Sc* extract and AHM treatments, indicating that the photosynthetic activity was changed by *Sc* and AHM treatments in chloroplasts of cotyledons, because both of positive and negative regulators are included in GO enrichment analysis. Intriguingly, two categories, “defense response” and “electron transport chain generation of precursor” were related to the genes increased and downregulated only in *Sc* treatment, as well as in the natural gall of *R. javanica*. These results suggest that not only phytohormones but also unknown effector molecules in the *Sc* extract regulated the gene expression of the Arabidopsis seedlings in this treatment.﻿

## Discussion

The wide variation in the unique shapes of insect galls has attracted attention. However, the molecular mechanisms underlying gall development remain poorly understood owing to the lack of a gall-inducing insect–host plant model. Gall-inducing insects produce effectors that can control the developmental programs of their host plants. In this study, we found that abnormal structures in the root tip region of *A. thaliana* induced by an extract of the gall-inducing aphid, *S. chinensis* have the typical characteristics of the insect gall structures^9–11^.

### Typical characteristics of the insect galls is induced by the treatment of *S. chinensis* extract

We found that the shape of the root region of *Arabidopsis* seedlings treated with the *Sc* extract significantly changed. The morphological changes in the root region of the seedlings can be characterized as follows: the essential elements of secondary cell wall (lignin, xylan, and suberin) were deposited in the epidermal cells of the root transition and elongation zone, indicating that secondary cell wall structures were constructed in the secondary cell wall-free cells of the root-transition zone . This characteristic appears to reflect the typical structure of the outer layer of gall tissue.

From the meristematic zone to the elongation zone, the width of the root epidermal cells thickened, while the parallel alignment of the cortical microtubule array was severely disrupted by *Sc* extract treatment. Furthermore, the bursiform and complex structures of vacuoles in the root division and transition zones changed to more fragmented, smaller vacuolar structures after *Sc* extract treatment. In addition, a stem cell niche marker (PLT1-GFP or PLT1-mNeonGreen and WOX5mNeonGreen fluorescence) was detected in the epidermal cells of the root-transition zone, in addition to the meristematic cells. From these results, we concluded that the cells of the root-transition zone became dedifferentiated and formed callus-like structures after *Sc* extract treatment. Furthermore, *Sc* extract treatment induced xylem vessel differentiation in the root-transition zone. The ability of TE differentiation was also confirmed by an in vitro xylem vessel formation induction system.

**Figure 8:**
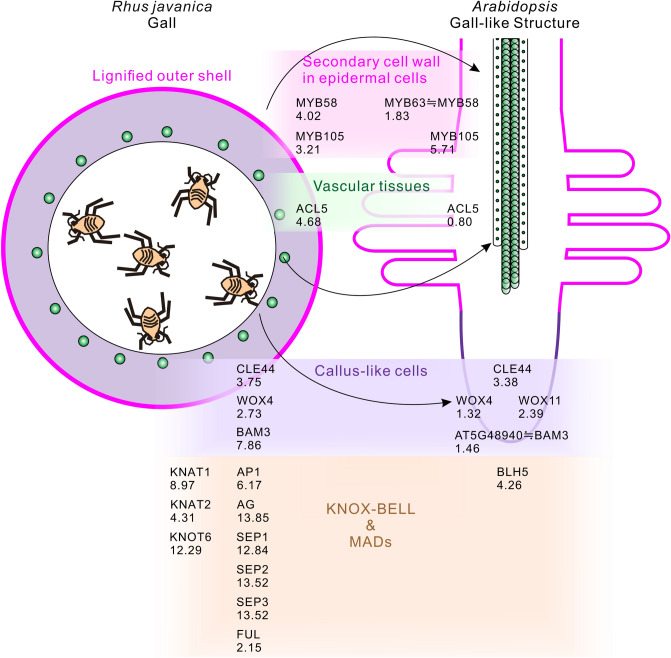
Comparison of the tissue structures and expressed genes of the gall-like structures in *Arabidopsis* in the root-tip region with *Rhus javanica* galls. The arrows in the schematic illustration indicate the corresponding tissue structures between *R. javanica* galls and *Arabidopsis* gall-like structures. The numbers in the figure represent the log-fold change values of the DEGs compared with each control tissue.

Collectively, we concluded that the *Sc* extract treatment could induce gall-like structures in the root tip region. The gall-like structures are composed of the secondary cell wall in the epidermal cells of the root elongation and differentiation zones, corresponding to the outer shells of galls; the enhancement of xylem vessel formation, corresponding to the newly formed vascular tissues inside galls; the dedifferentiation of epidermal and cortical cells in the root meristematic and elongation regions of the *Arabidopsis* seedlings, corresponding to the callus-like cells in galls (Fig. 8).

The gall-like structures induced only in the root-tip region are possibly because the cells in the root elongation zone still possess pluripotency. In fact, in root-knot nematode gall formation, the second-stage juvenile (J2) of the root-knot nematodes enters the inner root at the tip, and they migrates intercellularly to the vascular cylinder to reprogram root tissues of the root elongation zone into giant cells^35^. Moreover, the treatment with J2 crude extracts from outside of the seedlings induces abnormal structures in the root elongation zone^36^.

### Phytohormones in the aphid extract could partially induce gall-like structures in *Arabidopsis*

Phytohormones produced by gall-inducing insects play key roles in gall formation^27^. Active phytohormones such as IAA and CKs have been identified in several gall-inducing insects^17,27,28^. We previously identified amounts of IAA and CK in *S. chinensis*. In this study, in addition to IAA and CKs^6^, we observed abscisic acid, SA, and JAs inside the bodies of *S. chinensis*. Given that these phytohormones in insects are involved in the gall-like structure formation processes, including abnormal cell division, cellular enlargement, and dedifferentiation in *Arabidopsis* seedlings, the application of an AHM mimicking the phytohormone constituents of the *Sc* extract might contribute to the induction of the gall-like structures observed in the *Arabidopsis* seedlings. However, our results showed that the morphological changes (extra xylem vessel formation, the dedifferentiation of epidermal and cortical cells, and the formation of secondary cell wall in the epidermal cells), occurred only by the *Sc*-extract treatment in the root region of the *Arabidopsis* seedlings, suggesting the existence of effector molecules other than phytohormones in the *Sc* extract.

### Common characteristics of gene expression profiles of gall formation in *S. chinensis* and gall-like tissue formation in *Arabidopsis*

We found several commonly enriched the gene ontology (GO)-term categories of the genes expressed in the developmental gall of *Rhus javanica* and the gall-like tissue in *Arabidopsis* root-tip region. The corresponded GO-term categories are “response to hypoxia”, “defense response”, “phenylpropanoid metabolic process”, “response to oxidative stress”, response to osmotic stress”, “electron transport chain”, and “photosynthesis”. These categories are closely related to the typical characteristics of gall tissue of *R. javanica*^6^, e.g., the genes categorized to “phenylpropanoid metabolic process” is related to the synthesis of components of secondary cell wall of outer shell of gall tissue, “response to jasmonic acid” and “defense response” are related to the defense reaction of gall-tissue to the biotic stresses, and “response to hypoxia”, “response to oxidative stress”, “response to osmotic stress” are related to the biotic stress reaction of gall-tissue.

Our RNA-seq analysis of *Arabidopsis* seedlings after treating *S. chinensis* extract revealed that characteristic genes involved in the formation of the typical gall-structure are significantly increased. For instance, the stem cell maintenance genes, (*CLAVATA1*, *RGFR2*, *WOX4*, and *WOX5*), a suppressor of plant stem cell differentiation (*CLE44*), an essential factor for QC specification and stem cell activity (*PLT2*), root meristem growth factors required for root stem cell niche maintenance (*RGF1*-*RGF4*), the vascular development-related genes (*ACL5* and *VND7*), the genes involved in the secondary cell wall biosynthesis (*KNAT7*, *MYB105*, *MYB42*, *MYB63*, *SND2*, and *SND3*) were upregulated by the treatment using of *S. chinensis* extract (Table 2).

Collectively, we concluded that the expression profile of the gall-like structure induced by the *Sc*-extract treatment is similar to that of the developmental gall of *R. javanica* produced by *S. chinensis*.

## Conclusion

In this study, we demonstrated that gall-like structures can be induced in the root regions of *Arabidopsis* seedlings by the application of an extract of the gall-inducing aphid, *S. chinensis*. This phenomenon can be used as a model system to analyze the molecular mechanisms of gall formation processes. We referred to this model system as the *Arabidopsis*-based gall-forming assay (Ab-GALFA). The Ab-GALFA can be utilized as a bioassay for isolating the unknown insect effectors of gall formation in various host plants.

## Materials and methods

### Plant materials

The *A. thaliana* ecotype Columbia (Col-0) was used in this study. The transgenic marker lines used for the Ab-GALFA were as follows: *PLT1p::PLT1-GFP*^37^, *SYP22p::GFP-SYP22*^22^, *DR5rev::GFP*^33^, *TCS::GFP*^34^, and the microtubule marker line CaMV35Sp::GFP-fused microtubule binding domain (GFP-MBD)^21^. This study complies with relevant institutional, national, and international guidelines and legislation for using plant material.

### Plasmid construction and plant transformation

For the construction of *PLT1p::PLT1-GUS-mNeonGreen* and *WOX5p::WOX5-GUS-mNeonGreen* constructs, pGWB533 was digested with SacI, and then mNeonGreen PCR fragment, which was amplified from mNeonGreen vector using the following primers: (533-mNeon-N-f; 5’-cagcagggaggcaaacaaATGGTGAGCAAGGGCGAGGAGG-3′, and 533-mNeon-C-r; 5′-AACGATCGGGGAAATTCGTTACTTGTACAGCTCGTCCATGCC-3′), was ligated into SacI-digested pGWB533^38^ using NEBuilder HiFi DNA assembly kit (New England Biolabs, Ipswich, MA, U.S.A.) according to manufacturer’s instruction to generate pGWB533-GUS-mNeonGreen. The genomic fragments containing 5′-upstream region (1966 bp) plus the coding region of *PLETHORA1* (*PLT1*) or 5′-upstream region (1908 bp) plus the coding region of WUSCHEL RELATED HOMEOBOX 5 (WOX5) was then inserted into pGWB533-GUS-mNeonGreen via LR reaction (ThermoFisher Scientific, Waltham, MA, U.S.A.) to generate pGWB533-PLT1-GUS-mNeonGreen and pGWB533-WOX5-GUS-mNeonGreen, respectively. The transgenic *Arabidopsis* lines expressing PLT1-GUS-mNeonGreen or WOX5-GUS-mNeonGreen under control of each own-promoter were generated using *Agrobacterium tumefaciens* strain GV3101 by the floral dip method^39^.

### Growth conditions

The *Arabidopsis* seeds were sterilized with 70% ethanol and grown on half-strength Murashige and Skoog salt (1/2 MS) agar medium, supplemented with 1% sucrose, at 22 °C under a 16/8 h light/dark cycle. The 4-day-old seedlings were used for the Ab-GALFA.

### ***Arabidopsis***-based gall formation assay (Ab-GALFA)

Approximately 10 *S. chinensis* bodies isolated from the rapid growth phase of *R. javanica* gall, and 10 bodies of non-galling aphids *Acythosiphon pisum* and *Megoura crassicauda* were frozen in liquid nitrogen, ground to a fine powder, and suspended in 50 µL of sterile distilled water (DW), then the suspensions were centrifuged at 20,000 × *g* for 5 min at 4 °C to prepare the *S. chinensis* (*Sc*) extract stock solution. *Arabidopsis* seedlings grown vertically on 1/2 MS medium with 1.2% agarose for 4 days were gently removed from the agarose surface using micropipette tips, placed in a Petri dish, and the entire bodies of seedlings were incubated with different concentrations of *Sc*, *Ap* or *Mc* extracts or sterilized water (a negative control) under normal growth conditions for ≥ 15 h. Following incubation, the seedlings were used for subsequent analyses. The working solution of aphid extracts was prepared by diluting the stock solution.

### Quantitative analysis of various phytohormones in ***S. chinensis***

The endogenous levels of IAA, cytokinins, abscisic acid, and JAs in *S. chinensis* bodies were quantitatively analyzed as described by Yamane et al.^40^, with minor modifications. Briefly, aphid bodies (approximately 100–200 mg per sample) were collected, frozen in liquid nitrogen, and weighed. Then, the samples were ground and subjected to extraction in 80% acetonitrile and 1% acetic acid containing stable isotope-labeled compounds as internal standardsv[_13_C_6_-IAA, D_5_-tZ, D_6_-iP, D_6_-ABA, D_4_-SA (OlChemim, Olomouc, Czech Republic), D_5_-JA (CDN Isotopes, Quebec, Canada), and _13_C_6_-JA-Ile (kindly gifted by Dr. Yusuke Jikumaru, Riken, Japan; present affiliation: Agilent Technologies Japan, Ltd.)]. After purification of the HLB and MCX columns (Waters Corporation, Milford, MA, USA), phytohormones were analyzed using a 6410 Triple Quad LC/MS system (Agilent Technologies Inc., Santa Clara, CA, USA) equipped with a ZORBAX Eclipse XDB-C18 column and an XDB-C8 Guard column (Agilent Technologies). Peak areas were determined using MassHunter Workstation software (vB.04.00; Agilent Technologies). Four independent samples were analyzed to calculate the averages and standard deviations.

### Preparation of the phytohormone mixture solution

According to the results of the *S. chinensis* phytohormone content analyses*,* the concentrations in the Artificial Hormone Mixture (AHM) were as follows: 27.0 ng/mL IAA, 17.4 ng/mL ABA, 2.78 ng/mL SA, 0.43 ng/mL JA, and 0.086 ng/mL cytokinin in DW.

### Histological staining of ***Arabidopsis*** seedlings

The cell walls of the root cells were stained with propidium iodide, as previously described^35^. To stain the components of the secondary cell wall, the Arabidopsis seedlings were washed in sterilized water and incubated in 1 μg/mL WGA-Alexa Fluor 488 (ThermoFisher Scientific, Waltham, MA, U.S.A.) in sterilized water at 25 °C for 1 h and washed five times for 30 min for each wash. Lignin and suberin were stained with basic fuchsin and Auramine O, respectively, as described by Ursache et al.^31^.

### Image analysis

Confocal fluorescence and differential interference contrast images were obtained using a Leica TCS SP8 laser-scanning confocal microscope (Leica Microsystems, Wetzlar, Germany). The settings of excitation and emission for the observation of each fluorescent dye were as follows: propidium iodide, 555 nm/557–700 nm; GFP, 488 nm/493–547 nm; WGA-Alexa Fluor 488, 488 nm/493–547 nm; basic fuchsin, 552 nm/557–700 nm; and Auramine O 488 nm/493–547 nm. The captured images were processed using Leica LAS X software (Leica).

### Measurements of cell length, width, and microtubule density

After staining the root cells with propidium iodide, the length and width of the cells was determined using ImageJ software. The microtubule density in the GFP-TUB6-expressing line with or without the *Sc-*extract treatment was measured, as previously described^41^.

### Measurements of intensity and statistical analyses

Subsets of data with defined x-, y-, and z-dimensions were acquired using LASX software (Leica), and all subsets were transformed into 2D images using the maximum intensity projection function of LASX software. In all images, uniform brightness and contrast correction was performed before being exported for image analysis. All quantitative data were produced using the publicly available software Image Studio (LI-COR). The values were normalized relative to the average fluorescence intensity measured in the control group. Final statistical data evaluation and plot preparation were performed using R software.

### RNA extraction, library construction, and RNA sequencing

Total RNA was extracted from *Arabidopsis* seedlings using the RNeasy Plant Mini Kit (Qiagen, Hilden, Germany). All samples were pretreated with DNase I, using the Qiagen RNase-Free DNase Set (Qiagen, Hilden, Germany), to eliminate DNA contamination. RNA quality was checked by determining the RNA integrity number using an RNA 6000 bioanalyzer and RNA Nano Chip (Agilent Technologies). RNA-seq libraries were prepared using the Illumina TruSeq® Stranded RNA LT kit (Illumina, San Diego, CA, USA) according to the manufacturer’s instructions. Three independent RNA samples from each tissue were used for the analysis. Pooled libraries were sequenced using NextSeq 500 (Illumina), and single-end reads, 75 bp in length, were obtained. The obtained reads were mapped to the reference *A. thaliana* genome (TAIR10) using TopHat2^42^. The htseq-count script in the HTSeq library was used to count the reads^43^. Count data were subjected to trimmed mean of M-value normalization using EdgeR^44,45^. DEGs were defined using the EdgeR GLM approach^45^, and genes with FDRs < 0.05 were classified as DEGs. Scaled expression values were used for clustering, based on the SOM^46,47^.

### In vitro induction assay for xylem vessel formation

The xylem vessel cell differentiation assay have previously been described^32^*.* Briefly, *A. thaliana* (Col-0) seedlings were grown on MS solid medium containing 2.3 g/L MS salt mix (FUJIFILM Wako Chemicals, Osaka, Japan), 10 g/L sucrose, 0.5 mg/L nicotinic acid, 0.1 mg/L thiamine HCl, 0.5 mg/L pyridoxine HCl, 0.1 g/L myo-inositol, 2 mg/L glycine (pH 5.8), and 5 g/L phytagel (Sigma-Aldrich, St Lousis, MO, U.S.A. ) under dark conditions for 5 d after a 2-day-long vernalization period. Hypocotyls (25–30) were cut and transferred into 1-mL aliquots of TE-inducing medium (liquid MS medium) supplemented with 0.1 mg/L 2,4-D, 1 mg/L kinetin, and 10 µM bikinin, together with 0–10% (diluted with DW) *Sc* extract in 12 multi-well plates. The hypocotyls were cultured on a rotary shaker at 60 rpm for 8 days at 21 °C.

## Supplementary Information

Supplementary Information 1.

Supplementary Video 1.

Supplementary Information 2.

Supplementary Information 3.

Supplementary Information 4.

Supplementary Information 5.

## Data Availability

The datasets generated for this study can be found in the DDBJ as the BioProject Submission IDs; PRJDB13617 (https://ddbj.nig.ac.jp/resource/bioproject/PRJDB13617I) and PRJDB13618 (https://ddbj.nig.ac.jp/resource/bioproject/PRJDB13618). All data and materials appearing in this study are available from the corresponding author (M.H.S.) upon reasonable request.
