# Supplementary material for: Ab-GALFA, A bioassay for insect gall formation using the model plant Arabidopsis thaliana
Source: Sci Rep. 2023 Feb 13;13:2554. doi: 10.1038/s41598-023-29302-8 (PMC9925437; doi:10.1038/s41598-023-29302-8)
Supplement: Supplementary file 1 — Supplementary Information 1. [file 41598_2023_29302_MOESM1_ESM.pdf]

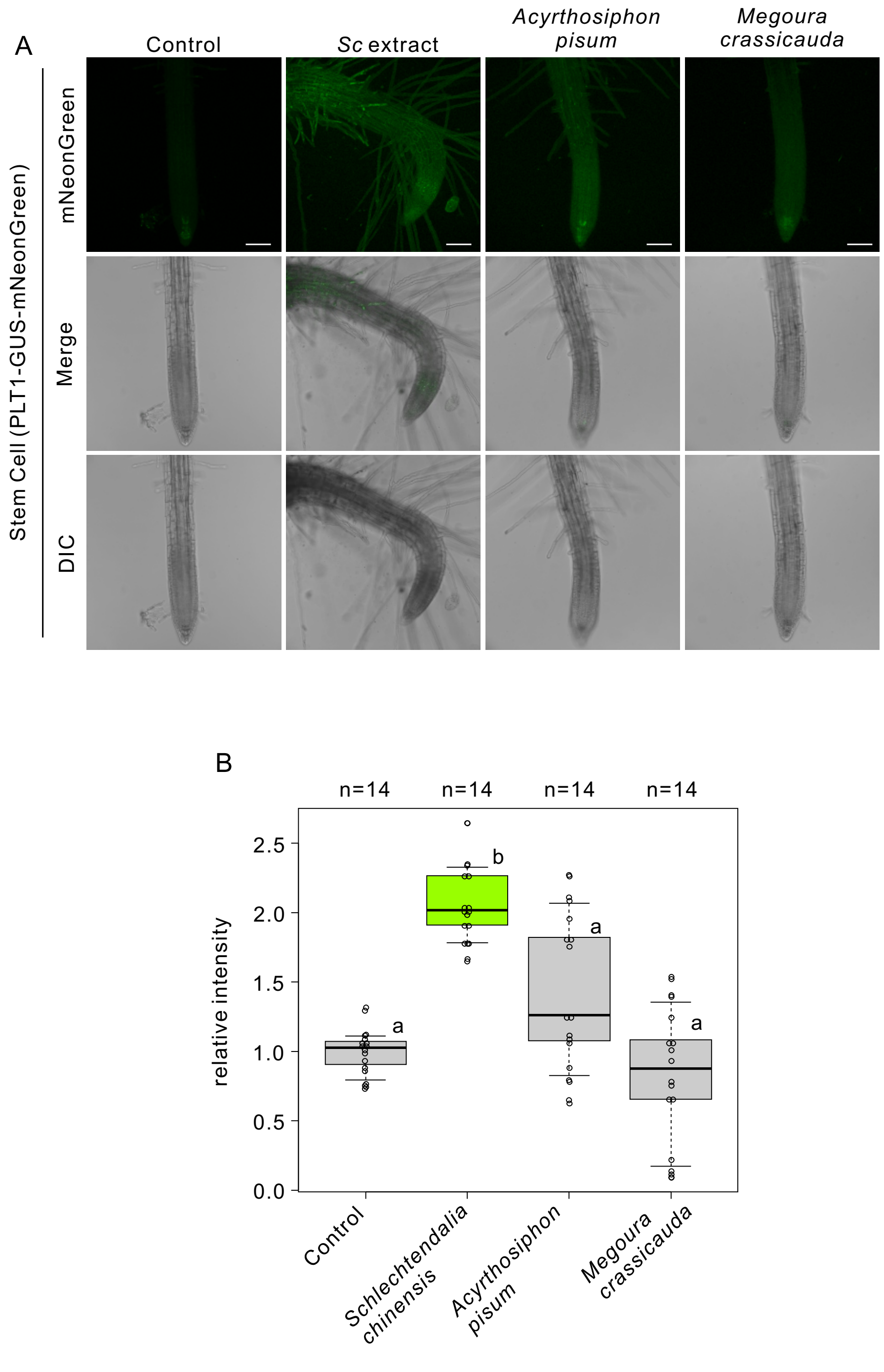

Supplementary Figure 1. Gall-inducing aphid extract initialized epidermal cells of root elongation zone. (A) Fluorescence images of *PLT1p::PLT1-GUS-mNeonGreen* expressing *Arabidopsis* line, treated with DW (control), *Schlechtendalia chinensis* (*Sc*), *Acyrthisiphon pisum* (*Ap*), *Megoura crassicauda* (*Mc*) extracts. Scale bars = 100 µm. (B) Box-and-whisker plots showing the fluorescence intensity in (A) (n = 14 seedlings for each treatment, with three biological replicates). The boxes and solid lines in the boxes show the upper (75th) and lower (25th) quartiles and median values, respectively. Groups with different letters are significantly different from each other ($p< 0.05$, Wilcoxon and Steel–Dwass tests). Different letters in (B) represent significant differences in each point ($p < 0.05$, Wilcoxon and Steel–Dwass test).

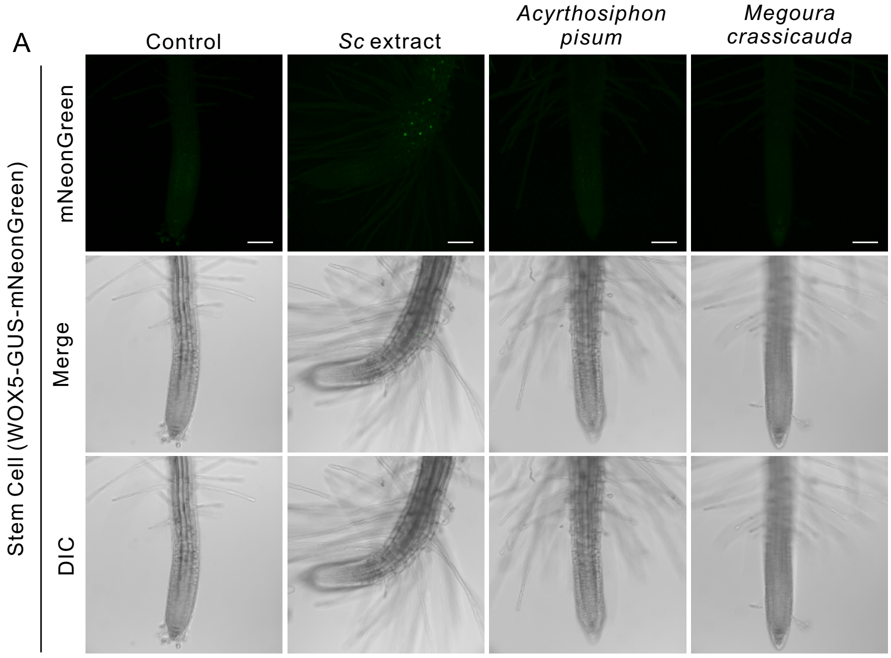

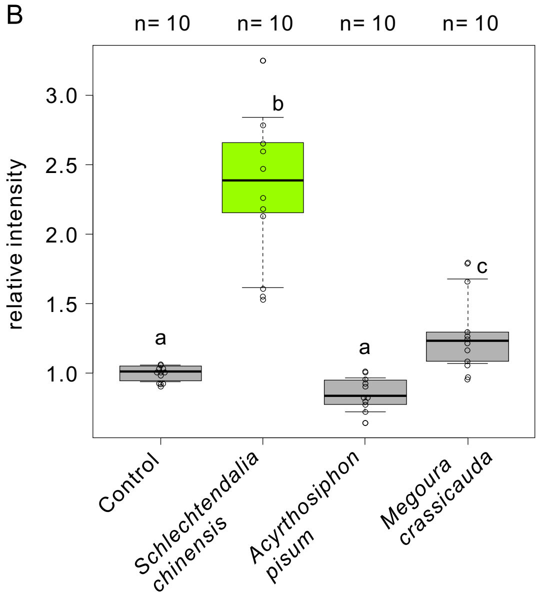

Supplementary Figure 2. Fluorescent images of a fluorescent marker of quiescent center. (A) Fluorescence images of *WOX5p::WOX5-GUS-mNeonGreen* expressing line, treated with DW (control), *Schlechtendalia chinensis* (*Sc*), *Acyrthisiphon pisum* (*Ap*), *Megoura crassicauda* (*Mc*) extracts. Scale bars = 100 µm. (B) Box-and-whisker plots showing the fluorescence intensity in (A) (n = 10 seedlings for each treatment, with three biological replicates). The boxes and solid lines in the boxes show the upper (75th) and lower (25th) quartiles and median values, respectively. Groups with different letters are significantly different from each other ($p < 0.05$, Wilcoxon and Steel–Dwass tests). Different letters in (B) represent significant differences in each point ($p < 0.05$, Wilcoxon and Steel–Dwass test).

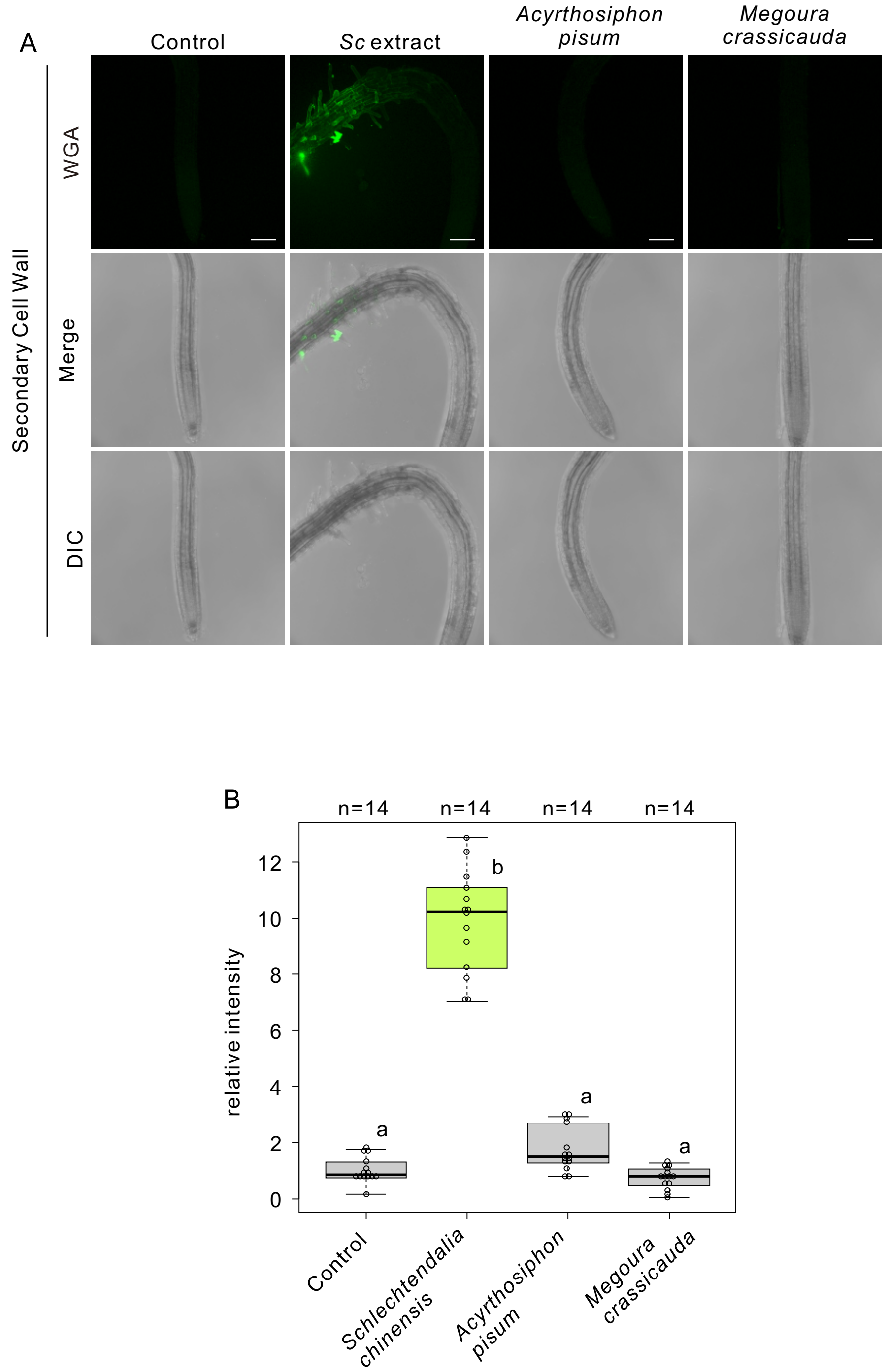

Supplementary Figure 3. *S. chinensis* extracts induced ectopic deposition of the secondary cell wall components in the epidermal cells of the root division zone. (A) Fluorescent and DIC images of 4-day-old *Arabidopsis* seedlings stained with Alexa Fluor 488-conjugated wheat germ agglutinin (WGA) for secondary cell wall labeling, treated with DW (control), *Schlechtendalia chinensis* (*Sc*), *Acyrthisiphon pisum* (*Ap*), *Megoura crassicauda* (*Mc*) extracts. Bars = 100 µm. (B) Box-and-whisker plots showing the fluorescence intensity in (A) (n = 10 seedlings for each genotype, with three biological replicates). The boxes and solid lines in the boxes show the upper (75th) and lower (25th) quartiles and median values, respectively. Different letters in (B) represent significant differences in each point ($p < 0.05$, Wilcoxon and Steel–Dwass test).

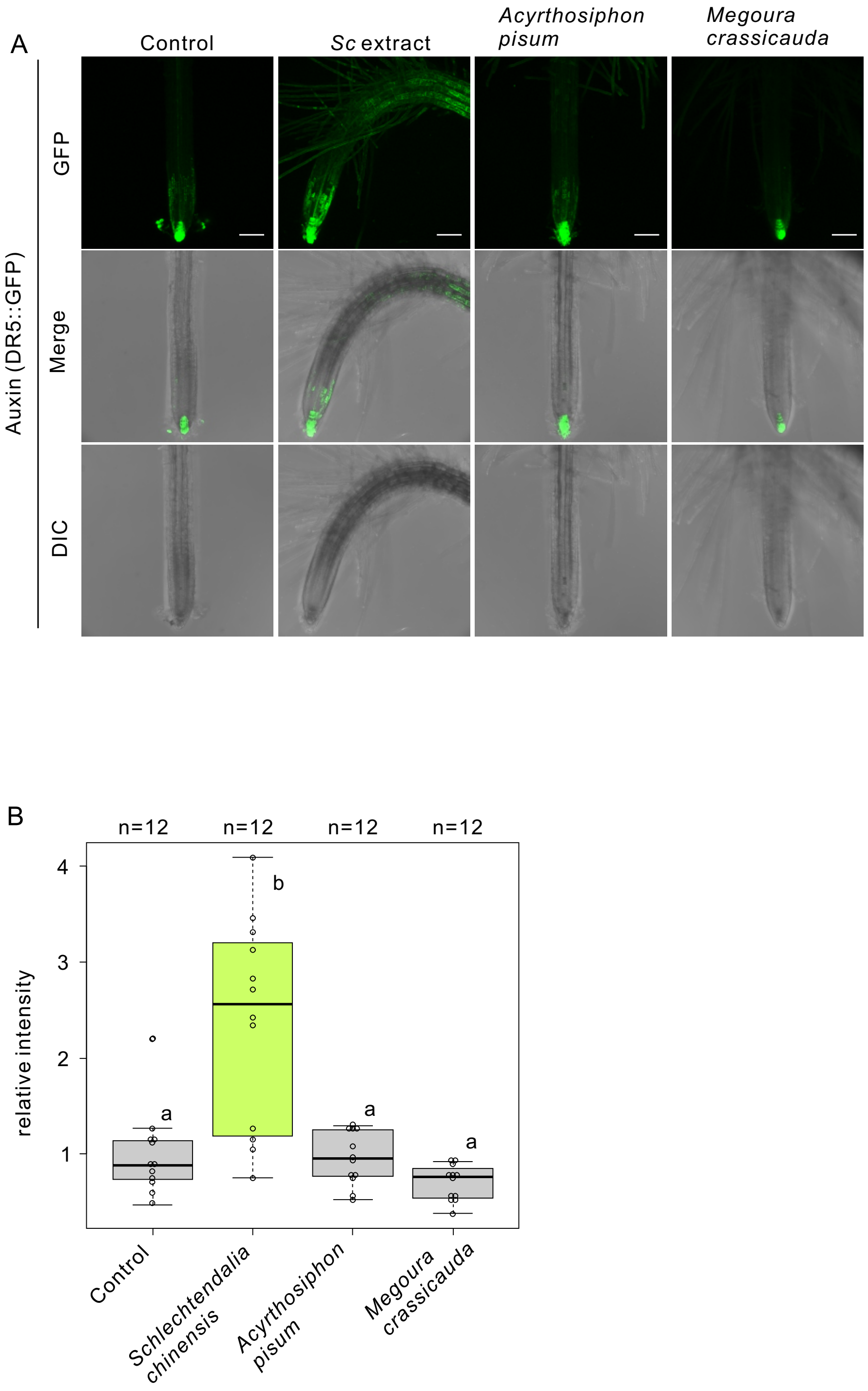

Supplementary Figure 4. ***S. chinensis*** **but not** ***A. pisum*** **and** ***M. crassicauda*** **extracts changes auxin distribution pattern in root.** (A) Fluorescent and DIC images of 4-d-old *Arabidopsis* seedlings of auxin fluorescence marker line, *DR5rev::GFP*, treated with DW (control), *Schlechtendalia chinensis* (*Sc*), *Acyrthisiphon pisum* (*Ap*), *Megoura crassicauda* (*Mc*) extracts. Bars = 100 µm. (B) Box-and-whisker plots showing the fluorescence intensity in (A) (n = 12 seedlings for each genotype, with three biological replicates). The boxes and solid lines in the boxes show the upper (75th) and lower (25th) quartiles and median values, respectively. Different letters in (B) represent significant differences in each point ($p < 0.05$, Wilcoxon and Steel–Dwass test).

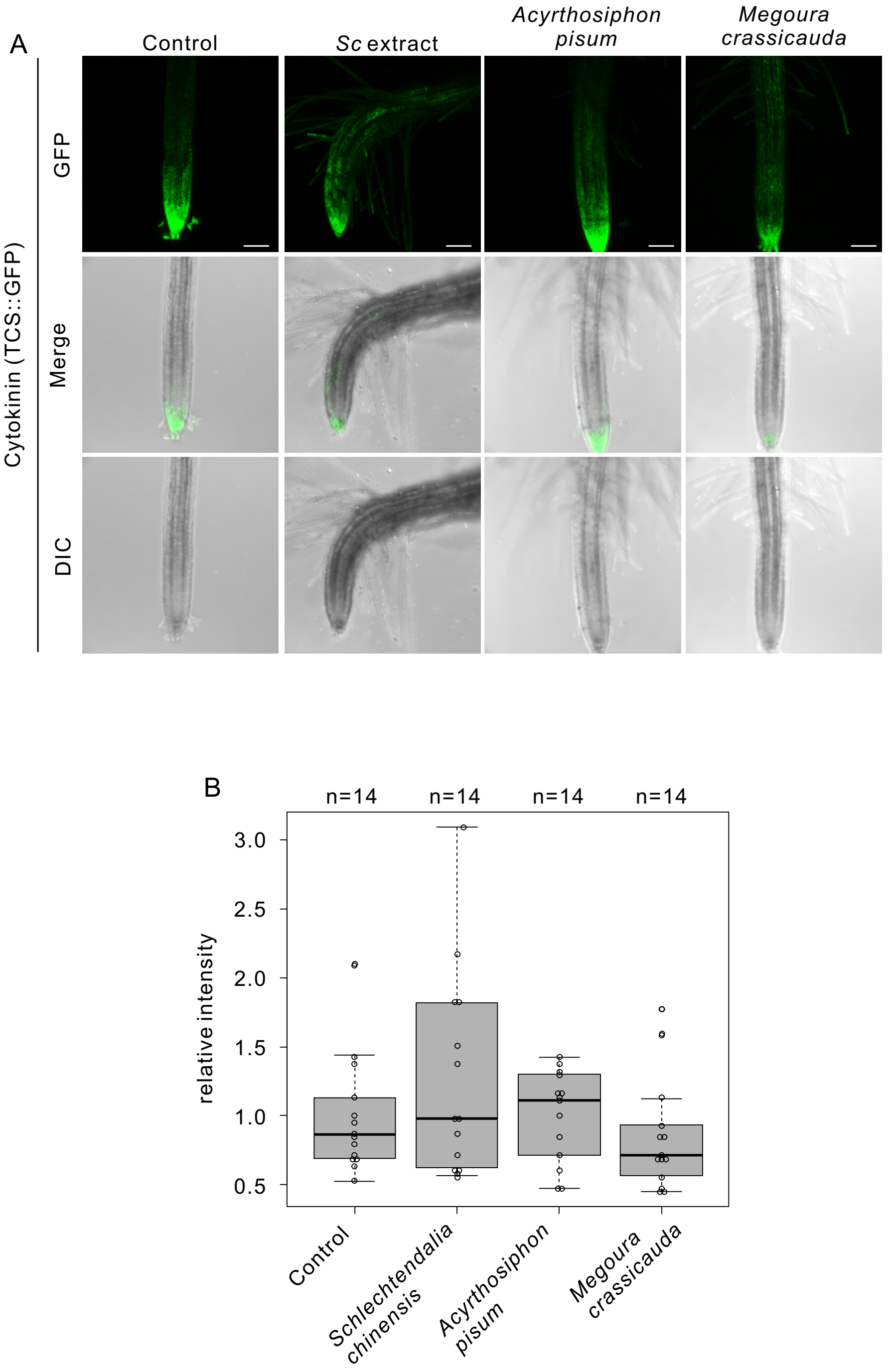

Supplementary Figure 5. Distribution of cytokinin is not altered in root by *S. chinensis* extract. (A) Fluorescent and DIC images of 4-day-old *Arabidopsis* seedlings of *TCS::GFP* auxin fluorescence marker line, treated with DW (control), *Schlechtendalia chinensis* (*Sc*), *Acyrthisiphon pisum* (*Ap*), *Megoura crassicauda* (*Mc*) extracts. Scale bars = 100 μm. (B) Box-and-whisker plots showing the fluorescence intensity in (A) (n = 14 seedlings for each genotype, with three biological replicates). The boxes and solid lines in the boxes show the upper (75th) and lower (25th) quartiles and median values, respectively. Different letters in (B) represent significant differences in each point ($p < 0.05$, Wilcoxon and Steel–Dwass test).
